# Optimization of Improved Motion-sensitized Driven-equilibrium (iMSDE) blood suppression for carotid artery wall imaging

**DOI:** 10.1186/s12968-014-0061-5

**Published:** 2014-08-09

**Authors:** Chengcheng Zhu, Martin J Graves, Jianmin Yuan, Umar Sadat, Jonathan H Gillard, Andrew J Patterson

**Affiliations:** 1 University Department of Radiology, University of Cambridge, Cambridge CB2 0QQ, UK; 2 Cambridge Vascular Unit, Cambridge University Hospitals NHS Foundation Trust, Cambridge CB2 0QQ, UK

**Keywords:** Carotid atheroma, Blood suppression optimization, Fast spin echo, Motion-sensitized driven-equilibrium

## Abstract

**Background:**

Improved motion-sensitized driven-equilibrium (iMSDE) preparations have been successfully used in carotid artery wall imaging to achieve blood suppression, but it causes notable signal loss, mostly due to inherent T_2_ decay, eddy current effects and B_1_^+^ inhomogeneity. In this study, we investigate the signal to noise ratio (SNR) and blood suppression performance of iMSDE using composite RF pulses and sinusoidal gradients. Optimized first moment (m_1_) values for iMSDE prepared T_1_- and T_2_- weighted (T_1_- and T_2_-w) imaging are presented.

**Methods:**

Twelve healthy volunteers and six patients with carotid artery disease underwent iMSDE and double inversion recovery (DIR) prepared T_1_- and T_2_-w fast spin echo (FSE) MRI of the carotid arteries. Modified iMSDE module using composite RF pulses and sinusoidal gradients were evaluated with a range of m_1_. SNR of adjacent muscle, vessel wall and the lumen were reported. The optimized iMSDE module was also tested in a 3D variable flip angle FSE (CUBE) acquisition.

**Results:**

The SNR of muscle was highest using sinusoidal gradients, and the relative improvement over the trapezoidal gradient increased with higher m_1_ (p<0.001). Optimal SNR was observed using an iMSDE preparation scheme containing two 180° composite pulses and standard 90° and -90° pulses (p=0.151). iMSDE produced better blood suppression relative to DIR preparations even with a small m_1_ of 487 mT*ms^2^/m (p<0.001). In T_1_-w iMSDE, there was a SNR decrease and an increased T_2_ weighting with increasing m_1._ In T_2_-w iMSDE, by matching the effective echo time (TE), the SNR was equivalent when m_1_ was <= 1518 mT*ms^2^/m, however, higher m_1_ values (2278 – 3108 mT*ms^2^/m) reduced the SNR. In the patient study, iMSDE improved blood suppression but reduced vessel wall CNR efficiency in both T_1_-w and T_2_-w imaging. iMSDE also effectively suppressed residual flow artifacts in the CUBE acquisition.

**Conclusions:**

iMSDE preparation achieved better blood suppression than DIR preparation with reduced vessel wall CNR efficiency in T_1-_w and T_2_-w images. The optimized m_1_s are 487 mT*ms^2^/m for T_1_-w imaging and 1518 mT*ms^2^/m for T_2_-w imaging. Composite 180° refocusing pulses and sinusoidal gradients improve SNR performance. iMSDE further improves the inherent blood suppression of CUBE.

## Background

Carotid atheroma is a known risk factor for subsequent stroke [1]. High resolution cardiovascular magnetic resonance (CMR) of the carotid artery allows for the visualisation and quantification of plaque composition and morphology and helps patient risk stratification [2]. Blood suppression is essential for black blood vessel wall imaging, and unsuppressed intra-luminal blood leads to plaque mimicking artifacts [3]. Double inversion-recovery (DIR) methods [4] have become the de facto standard for blood suppressed vessel wall imaging. However, this method is sensitive to the rate of flow replenishment which leads to plaque-mimicking artefacts in the presence of slow and turbulent flow that often occurs around the carotid bifurcation [3].

Motion-sensitized driven-equilibrium (MSDE) preparation has been proposed as an alternative blood suppression technique [5],[6]. An improved version of MSDE (iMSDE) has also been proposed which demonstrated better SNR performance [7],[8]. The blood suppression capability of iMSDE is not limited by the rate of flow replenishment, and it can suppress blood flow in every direction by applying flow sensitizing gradients along each axis. iMSDE has the potential to suppress turbulent and slow blood flow, which requires the prescription of higher m_1_ values. Furthermore, since iMSDE requires a shorter preparation time relative to DIR preparation, it is more time efficient.

One limitation of iMSDE preparation is that it causes notable signal loss, mostly due to T_2_ decay during the iMSDE preparation time. It is also sensitive to eddy current effects and B_1_^+^ non-uniformity [8]. Signal loss also becomes worse when high m_1_ values are prescribed [6].

T_1_- and T_2_-weighted (T_1_- and T_2_-w) double inversion recovery (DIR) prepared fast spin echo (FSE) sequences have been extensively used for plaque component characterization [2], however, carotid wall CMR with iMSDE preparation were rarely reported. Wang et al previously reported iMSDE prepared proton-density-weighted (PD-w) with m_1_ of 945 and 1524 mT*ms^2^/m [8]. Balu et al previously reported an iMSDE prepared 3D MERGE sequence with an m_1_ of 1524 mT*ms^2^/m [7]. However, to date there is a paucity of data comparing DIR and iMSDE to achieve optimal blood suppression and a lack of data on optimised iMSDE preparation timings for T_1_- and T_2_-w FSE protocols.

This study measures iMSDE induced signal intensity decay through phantom and volunteer studies. The optimized techniques were then tested on patients. These experiments were conducted across a range of first order moments (m_1_) used in the literature [8] and across T_2_ values known to reflect plaque composition [9]. Composite radiofrequency (RF) pulses are known to potentially reduce the signal loss due to B_1_^+^ non-uniformity [10], and sinusoidal gradients can potentially reduce eddy current effects. In this study, we report optimizations of SNR and blood suppression performance in iMSDE prepared carotid vessel wall CMR using composite RF pulses and sinusoidal gradients. DIR and iMSDE prepared T_1_ and T_2_-w protocols are then presented and compared in a group of normal volunteers and patients.

3D black blood variable flip angle FSE sequences are currently topical and have been applied to carotid imaging due to their intrinsic blood suppression ability and high SNR efficiency [11][12],[13]. However, residual artefacts can still appear at the carotid bifurcation, due to slow and complex flow [14]. In this study we briefly evaluate if the optimized iMSDE module can suppress these artefacts.

## Methods

### Study design

The experimental observations reported in this study were conducted on uniform test objects, calibrated gel phantoms, normal volunteers and asymptomatic patients. Both the volunteer and patient experiments were conducted following local ethical committee approval, all subjects involved gave informed written consent. The institutional review board of Addenbrooke’s hospital (Cambridge, UK) approved the whole study. The normal volunteers had the following demographics: n = 12, aged 25-50 years, 8 male. The patients had confirmed stenosis >30% and the following demographics: n = 6, mean age 65.7 years [range: 54 to 76 years], male = 3.

The test object experiments examined the image non-uniformity using permutations of standard and composite RF pulses, and compared theoretical and observed SNR change as a function of m_1_ using both trapezoidal and sinusoidal gradient types. The volunteer experiments measured sternocleidomastoid (SCM) muscle SNR changes using different RF pulse types, and also measured the intra-lumen and SCM muscle SNR change as a function of m_1_ and motion sensitizing gradient type. Optimizations for effective preparation regimes are reported for T_1_- and T_2_-w protocols by comparing conventional DIR preparations with iMSDE preparations with varying m_1_. The optimized iMSDE protocol was then validated on patients.

**Figure 1:**
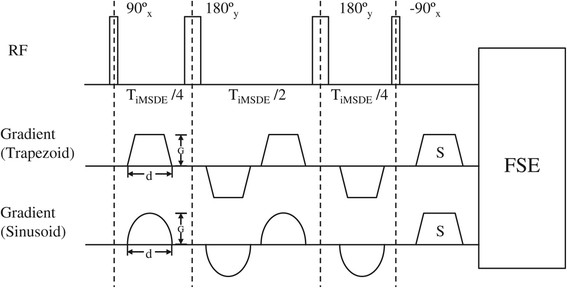
**Diagram of iMSDE preparation with trapezoid and sinusoid gradients.** Gradient amplitude (G) and duration (d) of the flow sensitive gradient are defined.

Pulse sequence diagrams of the iMSDE preparations used are shown in Figure 1. The specific parameters used to obtain the reported first order moments are listed in Table 1. The imaging experiments were conducted on a 1.5 T MR system (MR450, GE Healthcare, Waukesha, WI).

**Table 1:** **Parameters for iMSDE using sinusoidal and trapezoidal gradients with increasing first order moment (m**_**1**_**)**

**m**_**1**_**(mTms**^**2**^**/m)**	**Gradient strength (G) (mT/m) Trapezoidal/Sinusoidal**		**Gradient duration (d)**^**a**^**(ms)**	**Duration of preparation pulse**^**b**^**(ms)**
181	20	26	1.1	10.0
487	20	28	2.0	13.6
925	20	29	2.9	17.2
1518	20	30	3.4	21.0
2278	20	30	4.8	24.8
3108	20	30	5.7	28.4

^a^The duration of one gradient lobe.

^b^The duration from the start of the first 90° pulse to the end the -90° pulse.

### Phantom study

#### Composite RF pulses

A uniform cylindrical test object was scanned using a transmit/receive birdcage head coil. A PDw FSE sequence was prescribed with the following parameters: TR/TE_eff_: 3000 ms/8.8 ms; 16 cm × 16 cm field of view (FOV), 256 × 256 matrix, 5 mm slice thickness; number of excitations (NEX) = 1; echo train length (ETL) = 10. A single slice was prescribed through the middle of the phantom. iMSDE preparations were prescribed using variable RF pulse types including: no composite pulse (NOCP), composite -90° (CP90), composite 180° (CP180) and both composite 180° and -90° (CP180_90). Image non-uniformity was examined and compared between sequences. The use of a transmit/receive birdcage head coil allowed us to compare signal intensity variation as a function of the iMSDE preparation regime without dominant spatial location signal intensity variation as a result of RF reception (B_1_^-^) non-uniformity.

#### Motion sensitizing gradient and first order moment

Two gels with known T_2_ values of 50 ms and 89 ms respectively (Eurospin Test Object TO5, Diagnostic Sonar Ltd, West Lothian, Scotland, UK) were scanned using the same head coil. The gels were selected to best correspond with the reported T_2_ values of plaque [9] and SCM tissue [15],[16] found in vivo. Since no in vivo T_2_ measurement of SCM in 1.5 T was available, its T_2_ value was approximated using values from 0.5 T and 3 T [15],[16].

A PDw FSE sequence was prescribed with the following parameters: TR/TE_eff_: 3000 ms/8.8 ms; 24 cm × 24 cm FOV, 256 × 256 matrix, 5 mm slice thickness; NEX = 1; ETL = 10. A single slice was prescribed through the middle of the gels. iMSDE preparations were prescribed using trapezoidal and sinusoidal gradients with m_1_ values ranging from 181 to 3108 mT*ms^2^/m (defined in Table 1) using a composite 180° pulse. The experiment was then repeated to acquire a conventional PDw image without iMSDE preparation. The SNR of the gels was measured and compared between sequences.

#### Theoretical model

The theoretical model for T_2_ decay effects during the iMSDE preparation was calculated as $e^{-T_{\mathrm{iMSDE}}/T_{2}},$ where T_iMSDE_ is the time between the 90 and -90 RF pulses in the iMSDE module.

### Volunteer and Patient studies

Twelve healthy volunteers underwent DIR and iMSDE prepared, ungated, T_1_- and T_2_-w imaging of the carotid arteries using a 4-channel phased-array carotid coil (PACC, Machnet BV, Elde, The Netherlands). Scan parameters for the FSE sequence were: TR/TE (T_1_-w): 800 ms/10 ms; TR/TE_eff_ (T_2_-w): 2500 ms/50 ms; 14 cm × 14 cm FOV, 256 × 192 matrix, 3 mm slice thickness, NEX = 2; ETL (T_1_-w) = 10; ETL (T_2_-w) = 12, acquiring 4 slices to cover the bifurcation. A single and multi-slice DIR sequence was prescribed as defined previously [4]. Both iMSDE and DIR sequences acquired 1 slice per TR in the T_1_-w protocol and 4 slices per TR for the T_2_-w protocol. To offset the inherent T_2_ decay during the iMSDE preparation, the preparation time was included in the calculation of the effective TE, i.e. prescribed TE = TE_eff_ − T_iMSDE_. For example, in T_2_-w iMSDE acquisition with an m_1_ of 921 mT*ms^2^/m and a preparation time 17.2 ms, the prescribed TE was calculated as: 50 ms − 17.2 ms = 32.8 ms.

Six volunteers underwent iMSDE prepared T_1_-w FSE sequences using composite RF pulses and sinusoidal/trapezoidal gradients. Six separate volunteers also underwent combinations of DIR and iMSDE prepared T_1_- and T_2_- w imaging using sinusoidal gradients to determine optimal m_1_.

For validation purposes six patients were scanned with optimized settings for T_1_ and T_2_-w protocols.

To evaluate the iMSDE module within the 3D variable flip angle FSE sequence (CUBE, GE Healthcare, Waukesha, WI), a volunteer was scanned with and without iMSDE preparation with the following scan parameters: TR/TE: 440 ms/10 ms; 14 cm × 14 cm FOV; 224 × 224 matrix; 40 coronal slices; 1.2 mm slice thickness (interpolated to 0.6 mm); ETL 24. A 487 mT*ms^2^/m m_1_ and sinusoidal gradients were used for the iMSDE preparation.

#### Image analysis

For each carotid artery, 4 slices were selected for analysis. Measurements of SNR in the adjacent SCM muscle and within the lumen were performed using CMR tools (Cardiovascular Imaging Solutions, London, UK). In the six patients, the vessel wall SNR was also measured. Noise was determined as the standard deviation of the signal in an artefact free background region. SNR was calculated as:

(1) SNR=0.695×S/σ⋅

Where S is the signal intensity, σ ⋅ is the standard deviation of the noise, and the multiplier 0.695 corresponds to a four-channel coil correction [17]

Contrast to noise ratio (CNR) of muscle and wall was defined as:

(2) $${\mathrm{CNR}}_{\mathrm{eff},\;\mathrm{muscle}}\;=\left({\mathrm{SNR}}_{\mathrm{muscle}}-\;{\mathrm{SNR}}_{\mathrm{lumen}}\right)/\left(\sqrt{T_{\mathrm{sl}}}{\mathrm{SL}}_{\mathrm{th}}\right)\cdot$$

(3) $${\mathrm{CNR}}_{\mathrm{eff},\;\mathrm{wall}}\;=\left({\mathrm{SNR}}_{\mathrm{wall}}-\;{\mathrm{SNR}}_{\mathrm{lumen}}\right)/\left(\sqrt{T_{\mathrm{sl}}}{\mathrm{SL}}_{\mathrm{th}}\right)\cdot$$

Where T_sl_ ⋅ is the scan time (in minutes) per slice, and SL_th_ ⋅ is the slice thickness (in mm).

### Statistical analysis

Normality assumptions were formally assessed using a Shapiro-Wilk’s test. Distributions were summarised using the median [inter-quartile range]. An ANOVA was performed to test group differences between muscle and luminal SNR and CNR using the different composite RF preparations and also to test differences between sinusoidal and trapezoidal gradients. A Bonferroni correction was applied to account for the multiple comparison problem. Therefore, to account for the fact that 16 formal hypotheses have been investigated statistical significance was defined as p < 0.003125 i.e. 0.05/16. SNR and CNR comparison between groups was performed using non-parametric Wilcoxon signed-rank test. All the statistical analyses were performed using the statistical programming language R version 2.7.0 (The R Foundation of Statistical Computing, Vienna, Austria).

## Results

**Figure 2:**
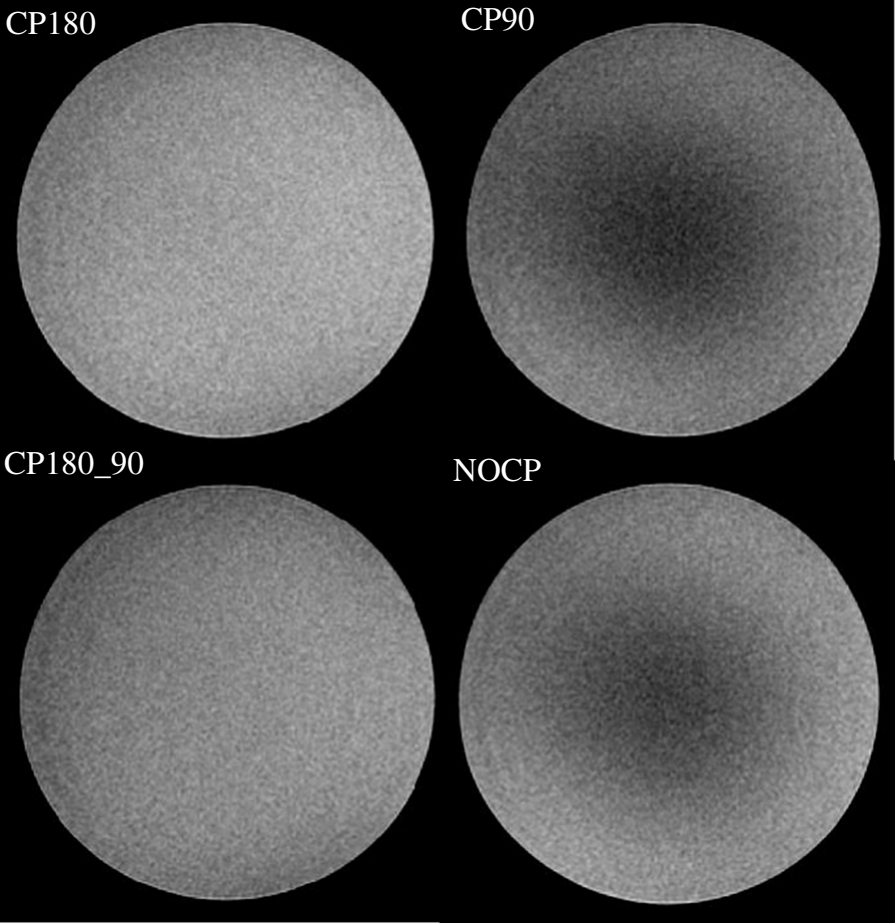
**PDw phantom images using varying iMSDE RF pulse types with identical windowing.** Composite 180° only (CP180), composite -90° only (CP90), composite 180° and composite -90° (CP180_CP90), and no composite RF pulses (NOCP). Sinusoidal gradients with a first order moment of 487 mT*ms^2^/m were used.

**Figure 3:**
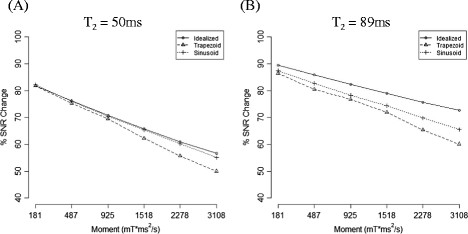
**Percentage SNR decrease using iMSDE relative to the standard sequence without blood suppression (in gel phantoms).** Comparison of SNR change is plotted against the idealized case (computing theoretical signal intensity change as a function of the increased effective echo time (TEeff)). Experimental observations of signal intensity change using trapezoidal and sinusoidal gradients for motion sensitization are illustrated using two calibrated gel phantoms with T2 values of 50 ms **(A)** and 89 ms **(B)** [1.5 T @ 21°C] to represent typical carotid vessel wall and muscle tissue respectively. A composite 180° pulse was used for all iMSDE preparations.

The test object study demonstrated that a composite 180° pulse improved the image uniformity, whereas the addition of a composite -90° pulse decreased the uniformity (Figure 2). We also observed that signal intensity decreased as a function of increasing first order moment m_1_ (Figure 3). T_2_ decay during T_iMSDE_ was responsible for the majority of the signal decrease. iMSDE using sinusoidal gradients demonstrated a better SNR performance compared to iMSDE using trapezoidal gradients with equivalent m_1_s, with this trend becoming more significant with increasing m_1_. This reduction was therefore attributed to eddy current effects. In addition, the signal drop was more significant in the gel with the shorter T_2_.

**Figure 4:**
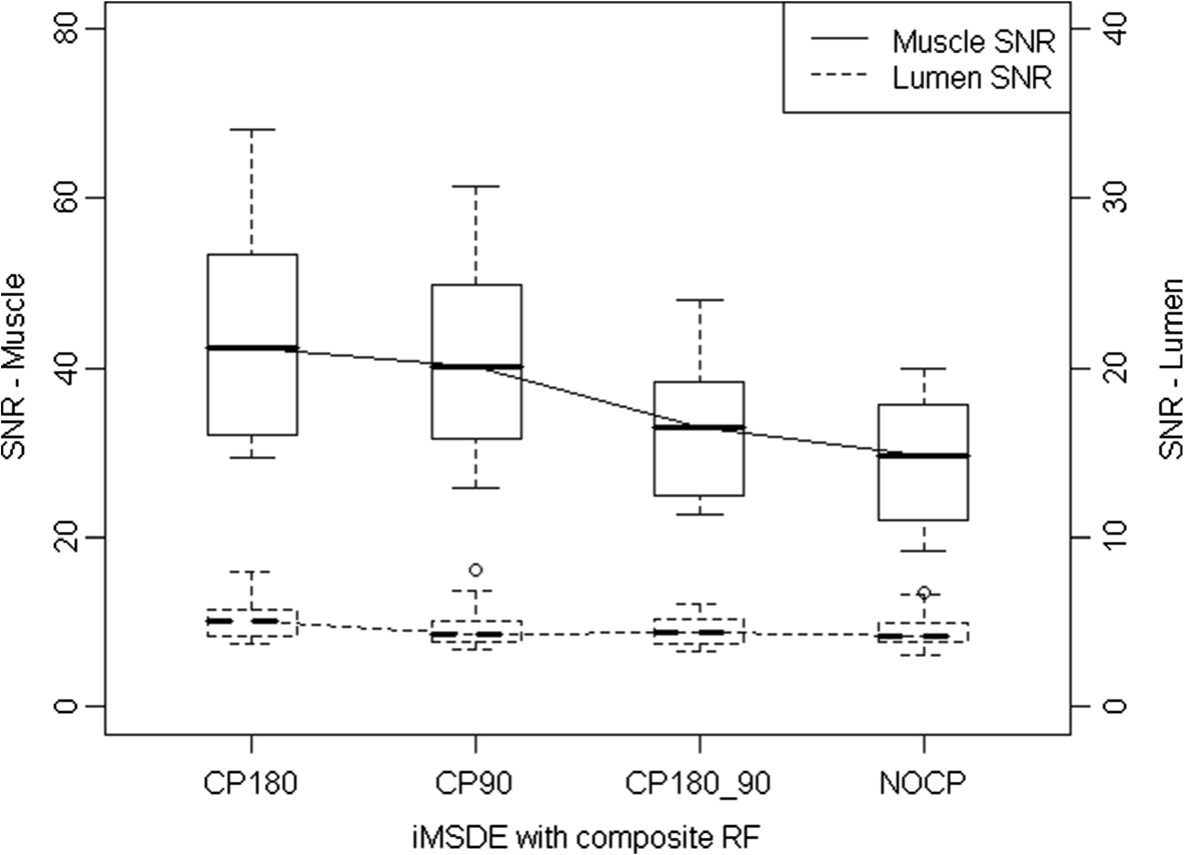
**Comparison of muscle and lumen SNR with varying iMSDE RF pulse type in T**_**1**_**-w images (6 volunteers, 48 locations).** Composite 180° only (CP180), composite -90° only (CP90), composite 180° and composite -90° (CP180_CP90), and no composite RF pulses (NOCP). Sinusoidal gradients with a first order moment of 487 mT*ms^2^/m were used.

In the volunteer study we observed that the composite 180° (CP180) yielded the best SNR performance (Figure 4 and Table 2). We noted that adding a composite -90° pulse reduced the SNR. Although the CP180 method appeared to provide the highest muscle SNR, we found no statistically significant SNR (p = 0.151) or blood suppression (p = 0.387) differences between RF preparations in vivo.

**Table 2:** SNR and CNReff comparison of the permutations of composite RF pulse types (six volunteers)

	**Muscle SNR median [IQ]**	**p-value**	**Lumen SNR median [IQ]**	**p-value**	**CNReff median [IQ]**	**p-value**
iMSDE						
CP180	50.2 [11.4]	0.151	5.4 [1.4]	0.387	20.9 [4.7]	0.154
CP180_90	44.7 [11.7]		4.9 [1.6]		19.2 [3.8]	
CP90	46.4 [9.7]		5.2 [1.5]		18.6 [4.8]	
NOCP	47.7 [12.6]		5.2 [1.4]		19.9 [5.3]	

iMSDE preparations were performed using a sinusoid gradient with a 487 mT*ms^2^/m first order moment.

**Figure 5:**
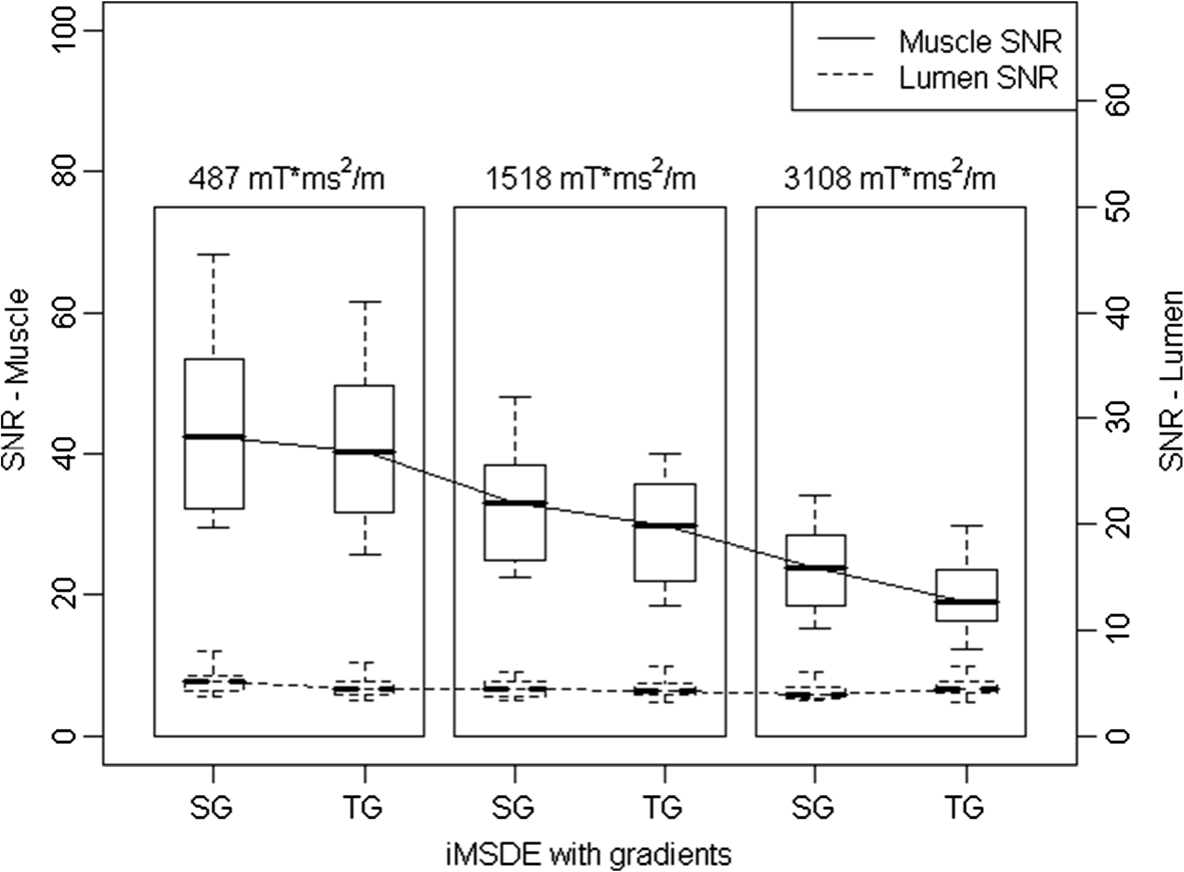
Comparison of muscle SNR change using iMSDE preparation with sinusoidal (SG) and trapezoidal (TG) gradients (6 volunteers, 48 locations).

T_1_-w iMSDE preparation using sinusoidal gradients showed better muscle SNR compared with trapezoidal gradients (Figure 5 and Table 3). This improvement was more significant with increasing m_1_ (6.5% higher SNR for 487 m_1_, 10.8% higher SNR for 1518 m_1_, 16.4% higher SNR for 3108 m_1,_ p < 0.001) (Table 3). There was no significant difference in blood suppression as a function of gradient type (p = 0.338).

**Table 3:** SNR and CNReff comparison of sinusoidal and trapezoidal motion sensitizing gradient with increased first order moment (six volunteers)

	**Muscle SNR median [IQ]**	**p-value**	**Lumen SNR median [IQ]**	**p-value**	**Muscle CNReff median [IQ]**	**p-value**
iMSDE						
Sinusoidal						
487 mT*ms^2^/m	50.2 [11.4]	<0.001*	5.4 [1.4]	0.338	20.9 [4.7]	<0.001*
1518 mT*ms^2^/m	37.1 [8.1]		4.4 [1.1]		15.3 [3.8]	
3108 mT*ms^2^/m	26.2 [8.1]		4.2 [1.4]		13.4 [3.4]	
Trapezoidal						
487 mT*ms^2^/m	48.8 [11.7]		4.8 [1.4]		20.5 [5.5]	
1518 mT*ms^2^/m	33.1 [7.3]		4.4 [1.1]		14.2 [4.9]	
3108 mT*ms^2^/m	21.9 [8.1]		4.7 [1.2]		10.3 [2.9]	

*: significant difference.

**Figure 6:**
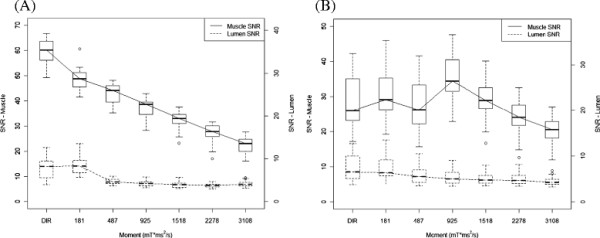
**iMSDE first moment optimization for T**_**1**_**-w (A) and T**_**2**_**-w (B) contrast (6 volunteers, 48 locations).**

**Figure 7:**
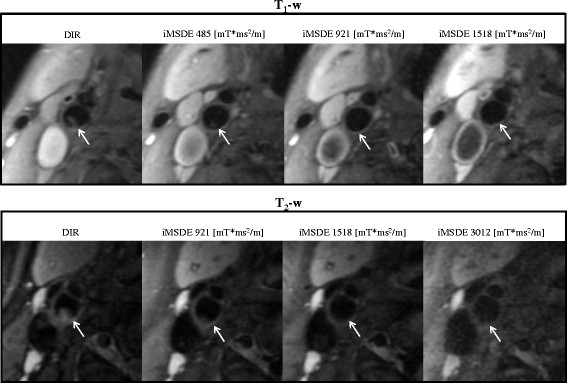
**Examples of iMSDE first order moment optimization in volunteers.** T_1_-w imaging with 485 mT*ms^2^/m and T_2_-w imaging with 1518 mT*ms^2^/m produced excellent blood suppression and SNR.

T_1_-w or T_2_-w iMSDE demonstrated better blood suppression compared with DIR (Figures 6 and 7) even with a small m_1_ value of 487 mT*ms^2^/m (p < 0.001). In T_1_-w iMSDE, the muscle SNR decreased with increasing m_1_. The lumen SNR decreased with the increased m_1_, but when the m_1_ was greater than 925 mT*ms^2^/m, no further improvement was observed. In T_2_-w iMSDE, by keeping the effective TE equivalent, no measurable SNR loss was found when m_1_ less than or equal to 1518 mT*ms^2^/m. Lumen SNR decreased with increased m_1_, but the improvement was negligible when m_1_ was greater than or equal to 1518 mT*ms^2^/m.

**Figure 8:**
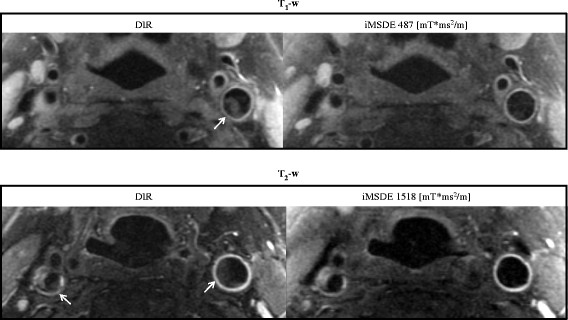
**A patient study using DIR and iMSDE prepared T**_**1**_**- and T**_**2**_**-w FSE.** Residual blood flow artefacts (arrows) observed following DIR preparation were suppressed using the optimized iMSDE module.

The optimized iMSDE prepared T_1_w and T_2_w protocols were applied in six patients. Good image quality was noted with better blood suppression (p < 0.001) with respect to the DIR method (Figure 8 and Table 4). In iMSDE T_1_w imaging, the SNR loss in muscle was 40.3%, whilst the SNR loss in the wall was 34.6%. In iMSDE T_2_w imaging, the SNR of muscle increased by 5.8% (due to the shorter effective TE) whilst the wall SNR decreased by 22.2%.

**Table 4:** Comparison of muscle and wall SNR/CNReff in patients using optimized iMSDE and DIR (six patients)

	**Muscle SNR median [IQ]**	**Wall SNR median [IQ]**	**Lumen SNR median [IQ]**	**Muscle CNR**_**eff**_**median [IQ]**	**Wall CNR**_**eff**_**median [IQ]**
T_1_w					
DIR	62.8 [17.3]	31.8 [10.0]	9.4 [5.0]	24.9 [5.7]	9.5 [6.3]
iMSDE 487 mT*ms^2^/m	37.5 [9.0]	20.8 [8.8]	5.4 [4.2]	15.0 [2.3]	6.5 [4.0]
p value	<0.001*	<0.001*	0.02*	<0.001*	<0.001*
T_2_w					
DIR	26.0 [7.8]	19.8 [11.5]	6.2 [3.1]	9.3 [2.2]	6.4 [4.8]
iMSDE 1518 mT*ms^2^/m	27.5 [6.5]	15.4 [10.5]	5.3 [1.7]	10.4 [2.2]	4.7 [5.0]
p value	<0.001*	0.01*	<0.001*	<0.001*	<0.001*

*: significant difference.

**Figure 9:**
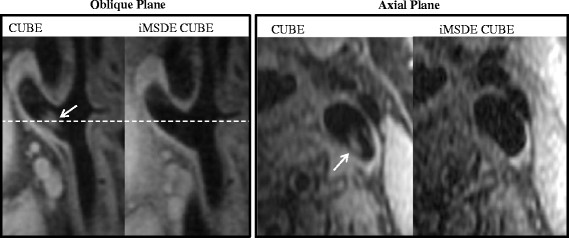
**Example images of 3D T**_**1**_**-w CUBE and iMSDE prepared CUBE images in a volunteer.** Blood flow artefacts (arrows) were further suppressed with the iMSDE preparation, however, a reduction in SNR was noted.

The optimized iMSDE module also removed flow artefacts in a T_1_w CUBE sequence but a reduction in image SNR was noted (Figure 9).

## Discussion

DIR prepared FSE sequences have been extensively used for carotid plaque imaging [2]. Wang et al previously reported that MSDE preparation achieved better blood suppression and higher muscle to lumen CNR efficiency than multi-slice DIR when using a T_2_-w FSE sequence [5]. Recently, Obara et al found 3D iMSDE prepared turbo field echo sequences had a higher muscle to lumen CNR efficiency than DIR prepared 2D FSE sequences for both T_1_-w and T_2_-w imaging in a group of five volunteers [18]. However, to date there have been no studies comparing the relative blood suppression and vessel wall CNR performance of iMSDE and DIR prepared FSE in generating the most clinically relevant T_1_- and T_2_- contrast weightings used for plaque imaging. Furthermore, the choice of an optimal first order moment (m_1_) within the iMSDE module is important, since m_1_ directly determines the blood suppression performance, and alters the image contrast and SNR.

In this study we demonstrated that a small m_1_ (487 mT*ms^2^/m) can achieve better blood suppression, albeit with reduced SNR in T_1_-w and T_2_-w FSE compared with conventional DIR preparation. We reported optimized m_1_ values to achieve blood suppression and SNR performance in both T_1_- and T_2_-w FSE sequences. In theory, blood suppression of iMSDE improves with increasing m_1_. However, a large m_1_ requires a longer preparation time that results in increased T_2_ decay and further signal loss due to the greater eddy current effects, hence causes an overall decrease in SNR. Therefore, for T_1_w contrast, m_1_ needs to be minimized in order to reduce T_2_ weighting and SNR loss. We found a small m_1_ (487 mT*ms^2^/m) can achieve satisfactory blood suppression, and at the same time minimize the SNR loss and T_2_ weighting (increasing the effective echo time by 13.6 ms) (Figures 6 and 7). Nonetheless, the considerable SNR loss (~40%) in T_1_w imaging can possibly limit its application in high-resolution imaging when SNR is low. When SNR is adequate, iMSDE can be used in T_1_w imaging to reduce plaque mimicking flow artefacts. Other novel blood suppression techniques, such as Delayed Alternating with Nutation for Tailored Excitation (DANTE), which induces smaller T_2_/T_1_ contrast changes with less SNR loss, may be optimal for T_1_w imaging [19]. Recently, Li et al. found DANTE has a higher vessel wall CNR efficiency than iMSDE and DIR methods in multi-contrast FSE imaging [20].

We found iMSDE preparation can achieve better blood suppression and better/comparable muscle SNR than DIR in T_2_-w imaging without affecting image contrast by matching the effective echo times with m_1_ < =1518 mT*ms^2^/m (Figures 6 and 7). Higher m_1_ values are not recommended as they lead to significant SNR loss without further improving the blood suppression.

As shown in the phantom study (Figure 3), plaque components with shorter T_2_ values than muscle would be expected to have even greater SNR loss due to the iMSDE preparation time. This hypothesis was evaluated in the patient sub-study.

In the volunteer study we found that the iMSDE preparation using a composite 180° RF pulse reduced the signal loss due to B_1_^+^ non-uniformity (Figure 4), which agreed with the test object results (Figure 2), but no significant differences were found (Table 2). Composite -90° did not appear to improve the image uniformity (Figure 2) and also resulted in a slight SNR decrease (Figure 4). This was due to the composite -90° slightly increasing the iMSDE preparation time by 2.4 ms (the composite -90° pulse comprises a -360° pulse and a 270° pulse with a total duration of 2.8 ms, while a simple -90° pulse has a duration of 0.4 ms).

The use of sinusoidal flow dephasing gradients reduced the signal loss presumed to be caused by eddy current effects in both the phantom and volunteer data. This trend became more obvious at higher m_1_ (Figure 5 and Table 2), possibly due to the increased eddy currents when larger flow-sensitizing gradients were applied. An intuitive advantage of applying sinusoidal gradients is that the dB/dt is relatively low which we would expect to lead to a reduction in eddy current induced artifacts [21].

Optimisation of arterial wall imaging using iMSDE blood suppression has potential clinical value, particularly for assessment of atheromatous plaques in the carotid artery. The carotid bifurcation is the most common anatomical site of atheroma formation. Due to irregular geometry and complex flow patterns particularly at higher grades of luminal stenosis, blood suppression can prove particularly challenging leading to plaque mimicking artefacts [3]. This may result in inaccurate measurements of plaque burden and luminal stenosis, both of which are clinically important criteria for refined severity assessment of carotid artery disease [22]-[24].

Plaque mimicking artefacts were frequently observed in our normal volunteer cohort (Figure 7). This may have been due to stagnant blood, which is common in subjects without luminal stenosis. This finding is also common in patients where medications such as ß-blockers may result in significant bradycardia. In our patient scans, blood flow artefacts were evident on both the T_1_w and T_2_w imaging, and the optimized iMSDE module successfully suppressed the flow artefacts without degrading the image quality (Figure 8).

Compared to DIR, iMSDE was found to provide a substantial improvement in blood suppression, thereby distinguishing the arterial lumen from the inner wall boundary and minimizing the likelihood of mistaking flow artefacts for wall thickening (Figures 7 and 8). Moreover, compared to trapezoidal flow dephasing gradients, the use of sinusoidal gradients resulted in an increase in muscle SNR (Figure 4) without compromising blood suppression. Also noteworthy is the fact that compared to DIR, iMSDE has a shorter preparation time (~20 ms) compared with DIR (>200 ms) [4]. The time efficiency of iMSDE preparation makes this technique more useful for vessel wall imaging than DIR preparation in multi-slice imaging.

Motion sensitivity of iMSDE may cause carotid wall signal loss due to pulsatility. However, the pulsatile motion of the carotid vessel wall during the cardiac cycle (around 1 s) is of the order of 0.4 mm [25], while the majority of the intra-luminal blood flow velocity is ~10 cm/s [26]. In the patient scan, we observed that in T_1_w imaging (using a small m_1_ of 487 mT*ms^2^/m), the percentage wall SNR decrease was less than the muscle SNR reduction (Table 4); whilst in T_2_w imaging (using a larger m_1_ of 1518 mT*ms^2^/m), the percentage wall SNR reduction was greater than the muscle SNR decrease. This is possibly due to the high m_1_ inducing signal loss in the wall due to pulsatile motion. However, even with a high m_1_, the signal loss in the vessel wall was still considered acceptable (~20%) and no obvious image quality degradation was observed.

In this study iMSDE was compared relative to the ungated multi-slice DIR preparation as proposed by Yarnykh and Yuan as the de facto standard [4]. Subsequent work has demonstrated that the ungated approach produces comparable SNR and plaque measurements whilst reducing scan time [27]. It is also important to consider that gated sequences will often produce variable image contrast as a result of variability in triggering.

3D variable flip angle FSE imaging of carotid plaque is gaining popularity for its intrinsic black blood effect, high isotropic resolution, large coverage and high SNR efficiency [11]-[13]. However in some situations of complex flow, it can still exhibit residual flow artefacts [14].

Previously MSDE has been used with a T_2_w SPACE (Siemens Healthcare, equivalent to CUBE) acquisition to improve the blood suppression [14]. Similarly, we have shown that our optimized iMSDE module can also improve the blood suppression in T_1_w CUBE. However, we did note a reduction in SNR. This was possibly a result of the shorter TR (440 ms) used in our T_1_w CUBE implementation. Since 3D FSE acquisitions such as CUBE and SPACE have an intrinsic black blood effect, a smaller m_1_ maybe sufficient for additional blood suppression if necessary. Future work will systematically investigate optimal blood suppression regimes appropriate for 3D CUBE.

## Conclusions

iMSDE with small m_1_ (487 mT*ms^2^/m) achieves better blood suppression but reduced vessel wall CNR efficiency in T_1_- and T_2_-w images relative to conventional DIR preparations. iMSDE is promising for T_2-_w imaging, however, we noted that it significantly reduced SNR and induced extra T_2_ weightings when applied in T_1_-w imaging. Optimized m_1_ for T_1_-w (487 mT*ms^2^/m) and T_2_-w (1518 mT*ms^2^/m) FSE are determined. The use of composite 180° refocusing pulses and sinusoidal gradients improves the SNR performance without compromising blood suppression efficiency. iMSDE is also suitable for 3D CUBE acquisitions and can further improve its inherent blood suppression.

## Abbreviations

iMSDE: Improved motion-sensitized driven-equilibrium

SNR: Signal to noise ratio

DIR: Double inversion recovery

FSE: Fast spin echo

RF: Radiofrequency

NEX: Number of excitations

ETL: Echo train length

FOV: Field of view

T_1_-w: T_1_-weighted

T_2_-w: T_2_-weighted

PD-w: Proton-density-weighted

## Competing interests

The authors declare that they have no competing interests.

## Authors’ contributions

CZ designed the study, developed the MR sequences, processed the data and wrote the manuscript; MJG developed the MR sequences and revised the manuscript; JY undertook image analysis tasks and revised the manuscript; US recruited the volunteers and patients and revised the manuscript; JHG processed the data and revised the manuscript; AJP designed the study, processed the data and revised the manuscript. All authors read and approved the manuscript.

## References

[B1] Lusis AJ Atherosclerosis Nature 2000 407 233 241 10.1038/35025203 11001066 PMC2826222

[B2] Underhill HR Hatsukami TS Fayad ZA Fuster V Yuan C MRI of carotid atherosclerosis: clinical implications and future directions Nat Rev Cardiol 2010 7 165 173 10.1038/nrcardio.2009.246 20101259

[B3] Steinman DA Rutt BK On the nature and reduction of plaque-mimicking flow artifacts in black blood MRI of the carotid bifurcation Magn Reson Med 1998 39 635 641 10.1002/mrm.1910390417 9543426

[B4] Yarnykh VL Yuan C Multislice double inversion-recovery black-blood imaging with simultaneous slice reinversion J Magn Reson Imaging 2003 17 478 483 10.1002/jmri.10278 12655588

[B5] Wang J Yarnykh VL Hatsukami T Chu B Balu N Yuan C Improved suppression of plaque-mimicking artifacts in black-blood carotid atherosclerosis imaging using a multislice motion-sensitized driven-equilibrium (MSDE) turbo spin-echo (TSE) sequence Magn Reson Med 2007 58 973 981 10.1002/mrm.21385 17969103

[B6] Koktzoglou I Li D Diffusion-prepared segmented steady-state free precession: application to 3D black-blood cardiovascular magnetic resonance of the thoracic aorta and carotid artery walls J Cardiovasc Magn Reson 2007 9 33 42 10.1080/10976640600843413 17178678

[B7] Balu N Yarnykh VL Chu B Wang J Hatsukami T Yuan C Carotid plaque assessment using fast 3D isotropic resolution black-blood MRI Magn Reson Med 2011 65 627 637 10.1002/mrm.22642 20941742 PMC3042490

[B8] Wang J Yarnykh VL Yuan C Enhanced image quality in black-blood MRI using the improved motion-sensitized driven-equilibrium (iMSDE) sequence J Magn Reson Imaging 2010 31 1256 1263 10.1002/jmri.22149 20432365 PMC2908521

[B9] Toussaint JF LaMuraglia GM Southern JF Fuster V Kantor HL Magnetic resonance images lipid, fibrous, calcified, hemorrhagic, and thrombotic components of human atherosclerosis in vivo Circulation 1996 94 932 938 10.1161/01.CIR.94.5.932 8790028

[B10] Brittain JH Hu BS Wright GA Meyer CH Macovski A Nishimura DG Coronary angiography with magnetization-prepared T2 contrast Magn Reson Med 1995 33 689 696 10.1002/mrm.1910330515 7596274

[B11] Jara H Yu BC Caruthers SD Melhem ER Yucel EK Voxel sensitivity function description of flow-induced signal loss in MR imaging: implications for black-blood MR angiography with turbo spin-echo sequences Magn Reson Med 1999 41 575 590 10.1002/(SICI)1522-2594(199903)41:3<575::AID-MRM22>3.0.CO;2-W 10204883

[B12] Mihai G Winner MW Raman SV Rajagopalan S Simonetti OP Chung YC Assessment of carotid stenosis using three-dimensional T2-weighted dark blood imaging: Initial experience J Magn Reson Imaging 2012 35 449 455 10.1002/jmri.22839 22147541 PMC4807862

[B13] Takano K Yamashita S Takemoto K Inoue T Sakata N Kuwabara Y Yoshimitsu K Characterization of carotid atherosclerosis with black-blood carotid plaque imaging using variable flip-angle 3D turbo spinecho: comparison with 2D turbo spin-echo sequences Eur J Radiol 2011 81 e304 e309 10.1016/j.ejrad.2011.10.012 22115798

[B14] Fan Z Zhang Z Chung YC Weale P Zuehlsdorff S Carr J Li D Carotid arterial wall MRI at 3 T using 3D variable-flip-angle turbo spin-echo (TSE) with flow-sensitive dephasing (FSD) J Magn Reson Imaging 2010 31 645 654 10.1002/jmri.22058 20187208 PMC2841222

[B15] Mavrogeni S Tzelepis GE Athanasopoulos G Maounis T Douskou M Papavasiliou A Cokkinos DV Cardiac and sternocleidomastoid muscle involvement in Duchenne muscular dystrophy: an MRI study Chest 2005 127 143 148 10.1378/chest.127.1.143 15653975

[B16] Biasiolli L Lindsay AC Chai JT Choudhury RP Robson MD In-vivo quantitative T2 mapping of carotid arteries in atherosclerotic patients: segmentation and T2 measurement of plaque components J Cardiovasc Magn Reson 2013 15 69 10.1186/1532-429X-15-69 23953780 PMC3751854

[B17] Constantinides CD Atalar E McVeigh ER Signal-to-noise measurements in magnitude images from NMR phased arrays Magn Reson Med 1997 38 852 857 10.1002/mrm.1910380524 9358462 PMC2570034

[B18] Obara M VAN Cauteren M Honda M Imai Y Kuroda K Assessment of Improved Motion-Sensitized Driven Equilibrium (iMSDE) for multi-contrast vessel wall screening Magn Reson Med Sci 2014 13 139 144 24769630 10.2463/mrms.2013-0036

[B19] Li L Miller KL Jezzard P DANTE-prepared pulse trains: a novel approach to motion-sensitized and motion-suppressed quantitative magnetic resonance imaging Magn Reson Med 2012 68 1423 1438 10.1002/mrm.24142 22246917

[B20] Li L, Chai JT, Biasiolli L, Robson MD, Choudhury RP, Handa AI, Near J, Jezzard P. **Black-Blood Multicontrast Imaging of Carotid Arteries with DANTE-prepared 2D and 3D MR Imaging.** *Radiology*. 2014; **131717**:ᅟ. 10.1148/radiol.14131717 24918958

[B21] Derek K. *Diffusion MRI: Theory, methods, and applications*. ᅟ:Oxford University Press; 2011.

[B22] European MRC Carotid surgery trial: interim results for symptomatic patients with severe (70-99%) or with mild (0-29%) carotid stenosis. European Carotid Surgery Trialists’ Collaborative Group Lancet 1991 337 1235 1243 10.1016/0140-6736(91)92916-P 1674060

[B23] Beneficial effect of carotid endarterectomy in symptomatic patients with high-grade carotid stenosis. North American Symptomatic Carotid Endarterectomy Trial Collaborators N Engl J Med 1991 325 445 453 10.1056/NEJM199108153250701 1852179

[B24] Bots ML Hoes AW Koudstaal PJ Hofman A Grobbee DE Common carotid intima-media thickness and risk of stroke and myocardial infarction: the rotterdam study Circulation 1997 96 1432 1437 10.1161/01.CIR.96.5.1432 9315528

[B25] Selzer RH Mack WJ Lee PL Kwong-Fu H Hodis HN Improved common carotid elasticity and intima-media thickness measurements from computer analysis of sequential ultrasound frames Atherosclerosis 2001 154 185 193 10.1016/S0021-9150(00)00461-5 11137099

[B26] Lal BK Hobson RW 2nd Tofighi B Kapadia I Cuadra S Jamil Z Duplex ultrasound velocity criteria for the stented carotid artery J Vasc Surg 2008 47 63 73 10.1016/j.jvs.2007.09.038 18178455

[B27] Mani V Itskovich VV Aguiar SH Mizsei G Aguinaldo JG Samber DD Macaluso FM Fayad ZA Comparison of gated and non-gated fast multislice black-blood carotid imaging using rapid extended coverage and inflow/outflow saturation techniques J Magn Reson Imaging 2005 22 628 633 10.1002/jmri.20428 16215965

